# Differential patterns of intronic and exonic DNA regions with respect to RNA polymerase II occupancy, nucleosome density and H3K36me3 marking in fission yeast

**DOI:** 10.1186/gb-2011-12-8-r82

**Published:** 2011-08-22

**Authors:** Brian T Wilhelm, Samuel Marguerat, Sofia Aligianni, Sandra Codlin, Stephen Watt, Jürg Bähler

**Affiliations:** 1 Department of Genetics, Evolution and Environment and UCL Cancer Institute, University College London, Darwin Building, Gower Street, London WC1E 6BT, UK; 2 Institut de Recherche en Immunologie et en Cancérologie (IRIC), 2900 boulevard Édouard-Montpetit, Montréal, H3C 3J7, Canada; 3 Salk Institute for Biological Studies, San Diego, CA 92186-5800, USA; 4 Cancer Research UK Cambridge Research Institute, Li Ka Shing Centre, Cambridge, CB2 0RE, UK

## Abstract

**Background:**

The generation of mature mRNAs involves interconnected processes, including transcription by RNA polymerase II (Pol II), modification of histones, and processing of pre-mRNAs through capping, intron splicing, and polyadenylation. These processes are thought to be integrated, both spatially and temporally, but it is unclear how these connections manifest at a global level with respect to chromatin patterns and transcription kinetics. We sought to clarify the relationships between chromatin, transcription and splicing using multiple genome-wide approaches in fission yeast.

**Results:**

To investigate these functional interdependencies, we determined Pol II occupancy across all genes using high-density tiling arrays. We also performed ChIP-chip on the same array platform to globally map histone H3 and its H3K36me3 modification, complemented by formaldehyde-assisted isolation of regulatory elements (FAIRE). Surprisingly, Pol II occupancy was higher in introns than in exons, and this difference was inversely correlated with gene expression levels at a global level. Moreover, introns showed distinct distributions of histone H3, H3K36me3 and FAIRE signals, similar to those at promoters and terminators. These distinct transcription and chromatin patterns of intronic regions were most pronounced in poorly expressed genes.

**Conclusions:**

Our findings suggest that Pol II accumulates at the 3' ends of introns, leading to substantial transcriptional delays in weakly transcribed genes. We propose that the global relationship between transcription, chromatin remodeling, and splicing may reflect differences in local nuclear environments, with highly expressed genes being associated with abundant processing factors that promote effective intron splicing and transcriptional elongation.

## Background

Generation of mature mRNA transcripts requires complex and interconnected processes that involve opening of the local chromatin structure around the DNA region to be transcribed, binding and transcription by RNA polymerase II (Pol II), and processing of the pre-mRNAs, including the splicing of the non-coding introns [1,2]. Protein production is streamlined at several levels of gene expression, including coordinated transcription and translation [3]. Moreover, there is some evidence for functional coupling between transcription and pre-mRNA processing [4-6].

We have previously reported that, in fission yeast (*Schizosaccharomyces pombe*), highly transcribed genes tend to be most efficiently spliced while lowly transcribed genes are less efficiently spliced [7]. The reason for this unexpected global coordination between transcription and splicing is not known. Moreover, Pol II-directed transcription is controlled by permissive or repressive chromatin modifications but in turn also affects such modifications [8]. Splicing is initiated co-transcriptionally in a chromatin context, which raises the possibility of a functional relationship between splicing and the local chromatin environment. In addition to controlling the accessibility of DNA to the basal transcriptional machinery, there is evidence that chromatin structure can influence the co-transcriptional splicing of immature transcripts [9-11]. Notably, differential marking of introns and exons has recently been reported in several organisms [12,13], although the mechanism and functional consequences of such marking are not clear.

We applied multiple genome-scale approaches in fission yeast to clarify the relationships between chromatin, transcription and splicing. Introns, besides promoter and terminator regions, were relatively depleted of histones and also showed distinct chromatin patterns. Unexpectedly, Pol II occupancy was much higher in intronic than in exonic DNA regions, most notably in lowly expressed genes. This differential marking of introns at the DNA level suggests that Pol II stalls at the 3'-ends of intronic regions, leading to substantial accumulation in the introns of lowly transcribed genes. We speculate that these patterns reflect a functional coupling between transcription, chromatin remodeling, and splicing, and that only highly transcribed genes are embedded in processive environments such as 'transcription factories', where abundant processing factors promote effective intron splicing and transcriptional elongation.

## Results and discussion

### Experimental approach

In order to uncover any connections between transcription, intron splicing, and chromatin marks in rapidly growing fission yeast cells, we determined global Pol II occupancy using chromatin immunoprecipitation on microarray (ChIP-chip) experiments. Furthermore, we applied ChIP-chip experiments to analyze the global distributions of histone H3 and lysine 36 trimethylation of histone H3 (H3K36Me3), a modification that is enriched in the body of actively transcribed genes [14]. In addition, to verify the histone H3 occupancy and reveal genomic regions that are relatively protein free, we applied formaldehyde-assisted isolation of regulatory elements (FAIRE) [15,16]. We used the same high-density Affymetrix tiling array platform for all these genome-wide approaches (Materials and methods).

### Distinct Pol II occupancy and chromatin patterns in promoter and terminator regions

**Figure 1:**
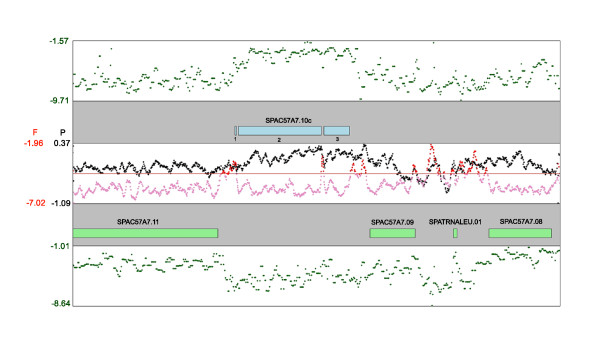
**An example of FAIRE, Pol II ChIP-chip, and expression data**. The top and bottom panels with green points depict expression data for the upper and lower strand, respectively, obtained from random-primed RNA hybridized to Affymetrix tiling arrays with each point representing a single probe. The second and fourth panels show annotated genes in the region around *sec21 *(SPAC57A7.10c), with exons numbered underneath the gene. The third panel shows a 5 probe running average of Pol II signals (black points) or FAIRE signals (pink/red points). The horizontal red line shows the 85% percentile line for all FAIRE probe signals, with probes above this cut-off colored red and those below colored pink. Note that FAIRE and Pol II signals are not strand-specific.

**Figure 2:**
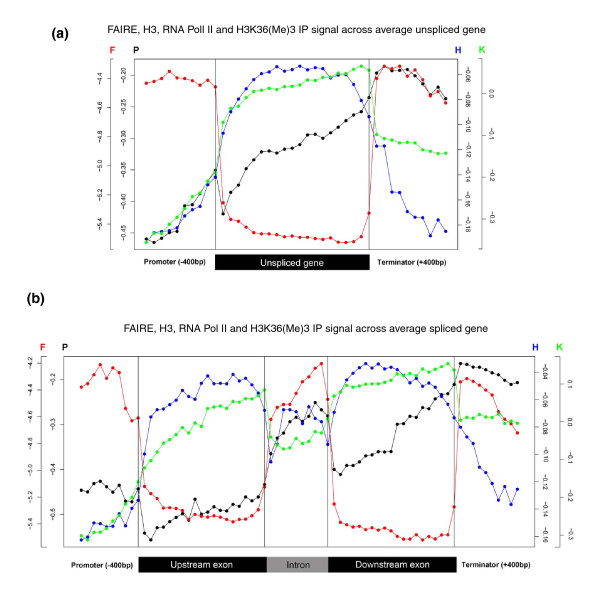
**Profiles of transcription- and chromatin-related patterns across average spliced and unspliced genes**. **(a) **Average unspliced gene profiles for FAIRE (red), histone H3 (blue), H3K36me3 (green, normalized for H3 signals), and Pol II (black) signals from Affymetrix tiling arrays. Promoter and terminator regions are taken as 400 bp up- and downstream of the start and stop codons, respectively, and divided into 10 bins of 40 bp each, while the coding regions were divided into 20 bins of equal size. Black vertical lines separate different gene sections, and each plotted point represents the average of all probes that fall into the respective location bin. Color-coded scales for FAIRE (F) and Pol II (P) signals are shown on the left y-axis of the graph, while the scales for histone H3 (H) and H3K36me3 (K) are shown on the right y-axis. **(b) **Average spliced gene profiles for FAIRE (red), histone H3 (blue), H3K36me3 (green), and Pol II (black) signals from Affymetrix tiling arrays as in (a).

The 5' ends of genes, corresponding to the nucleosome-free regions of promoters, had high FAIRE signals in fission yeast (Figure 1). These results are consistent with the originally published results in human [15]. Figure 2 shows the average patterns for the different chromatin- and transcription-related features across intron-less and intron-containing genes. Peaks of Pol II enrichment were evident in the promoter regions of genes, reflecting the accumulation of Pol II before transcription elongation [17,18]. Moreover, these regions showed high FAIRE signals, but relative depletion of histone H3 and, even more so, for its H3K36Me3 modification (Figure 2).

**Figure 3:**
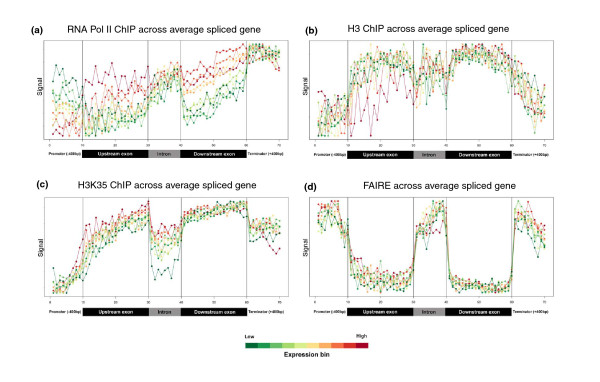
**Profiles of transcription and chromatin-related patterns as a function of gene expression**. **(a-d) **Probe signals for Pol II (a), histone H3 (b), H3K36me3 (c), and FAIRE (d) were used to generate average spliced gene profiles that were grouped into ten ranked bins based on Affymetrix expression data. Scales for the relative data range from each expression bin were used to generate the plots. Identical data plotted on the same absolute y-scale for all expression bins is presented for average spliced and unspliced genes in Figure 4. The color bar at bottom depicts average expression levels of bins (red, high expression; green, low expression), and black vertical lines within each box demarcate different sections within the average gene.

Gene promoters are known to contain nucleosome-free regions [19-21]. Notably, we found that the 3' ends of genes, corresponding to the terminator regions, also show Pol II enrichment, low histone H3 density and high FAIRE signal (Figures 2 and 3). While the nucleosome-free regions in promoters have been well characterized, a similar depletion of nucleosomes in terminator regions is not as well defined. A recent report in budding yeast shows depletion of nucleosomes at the 3' end of transcribed genes, and this depletion is coupled to transcriptional activity [22]. Our findings are also consistent with reports in mammalian cells that describe pausing of Pol II in terminator regions [23,24]. The start and end of introns showed lower levels of H3 occupancy (Figure 2b). This pattern might result from a 'looped' arrangement of exons and introns analogous to that proposed for the human *BRCA1 *gene [25]. Although this exon-intron pattern is not reflected in FAIRE, the overall patterns support the notion that nucleosome density is likely the major determinant for the FAIRE signals.

### Gene expression levels affect Pol II occupancy and chromatin patterns across genes

**Figure 4:**
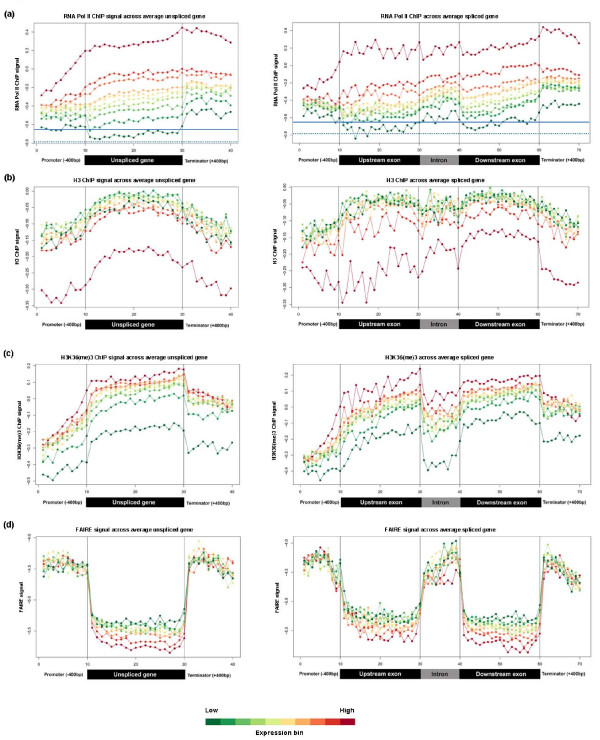
**Profiles of transcription and chromatin-related patterns as a function of gene expression**. **(a-d) **Probe signals for Pol II **(a)**, histone H3 **(b)**, H3K36me3 **(c)**, and FAIRE **(d) **were used to generate average spliced gene profiles that were grouped into ten ranked bins based on Affymetrix expression data. Average values for each bin within each expression group were plotted on the same absolute scale for each experiment type. For panel (a), the background level of RNA Pol II enrichment was estimated by calculating the average signal from all probes (152,253) that fell outside of binned regions for analysis. This background average is shown as a horizontal blue solid line. Because some atypically large untranslated regions and novel annotated regions will also contribute signal to this value, a second average (horizontal blue dotted line) is shown where the top 10% of probes by signal (15,226) are removed. The red-to-green color bar at the bottom of the figure depicts average expression levels of bins (red, high expression; green, low expression), and black vertical lines within each box demarcate different sections within the average gene.

We next assessed the effects of transcript levels on the observed Pol II- and chromatin-related patterns across genes. To this end, we sorted all genes with measurable expression on Affymetrix chips into decile ranked groups, with the first decile representing the 10% most highly expressed genes, and so on. Average expression values for unspliced and spliced genes were calculated for each data set and for each expression bin and plotted either relative to the values in each bin (Figure 3) to highlight the range within each expression group or on a single scale according to the range of values of the entire dataset (Figure 4) to show the absolute enrichment. This analysis revealed that gene expression levels strongly influence the Pol II- and chromatin-related patterns. Coherent differences depending on expression level group were apparent (Figure 4): the most highly expressed genes showed the highest Pol II occupancy (Figure 4a), but the lowest density of histone H3 (Figure 4b), and the highest levels of H3K36me3 modification (after correcting for nucleosome density; Figure 4c). Glover-Cutter *et al*. [26] made similar observations of inverse enrichment between Pol II and nucleosomes, which could reflect displacement of nucleosomes by Pol II. The Pol II patterns were also apparent at the level of highly or lowly expressed single genes (Additional file 1).

Expression level-dependent differences in Pol II promoter patterns were also apparent: only lowly expressed genes showed promoter regions with higher levels of Pol II enrichment relative to downstream exonic regions, while highly expressed genes had more Pol II in exonic regions (Figure 3a). While Pol II is known to pause on promoters of some genes [17,18], a global and gradated relationship between gene expression level and Pol II enrichment at promoters has not been reported previously. Unlike the promoter-associated accumulation, the increased Pol II occupancy at the terminator region showed little difference with varying expression levels (Figure 3). Our data show that Pol II behavior in terminator regions is less dependent on expression level than in promoter regions. The enrichment of Pol II in the terminator region could reflect the time required for the release of Pol II from the DNA and/or interactions between promoter and terminator regions [25,27,28].

### Distinct chromatin patterns in intronic regions

**Figure 5:**
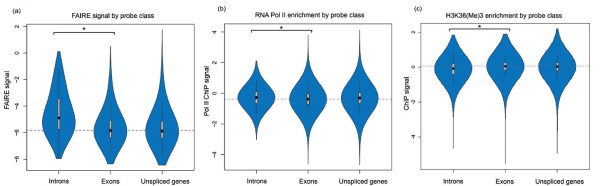
**Violin plots of FAIRE and Pol II signals**. **(a-c) **Violin plots (combining box plot and kernel density plot) show the uni-modal distribution of signals for probes entirely within introns and exons (spliced genes) or entirely within coding regions (unspliced genes) for FAIRE **(a)**, Pol II occupancy **(b)**, and H3K36me3 ChIP-chip signals **(c)**. The median signal for probes in exons is shown by the dashed horizontal line. Signal differences (shown on the y-axis) between introns and exons (indicated by the bar and asterisk) are significantly different (*P*-value < 2.2 × 10^-16^; Welch two sample *t*-test).

**Figure 6:**
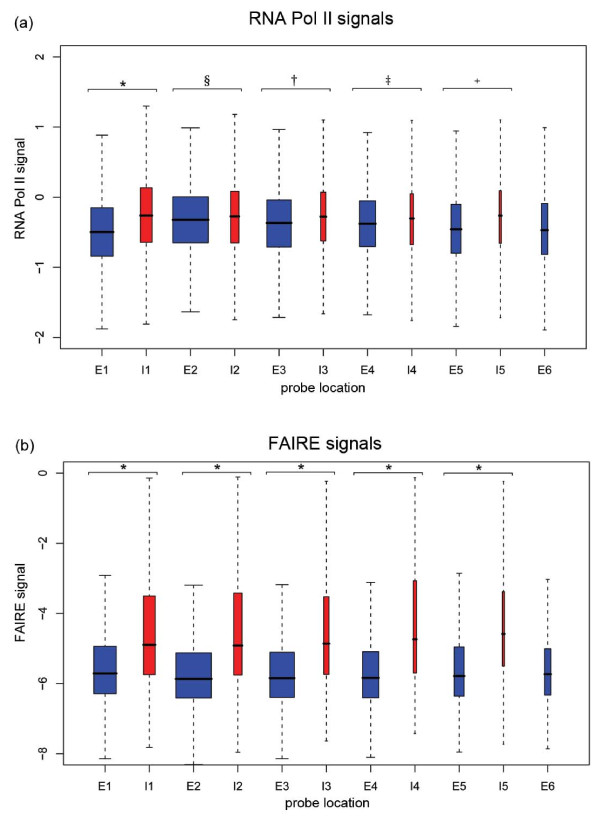
**FAIRE/Pol II occupancy signals for introns and exons by position**. **(a, b) **Box plots showing signal distributions for Pol II occupancy **(a) **and FAIRE **(b) **in spliced genes by exon or intron number (E1 for exon1, I1 for intron1, and so on). The average signal for each intron position was compared to the average signal for each previous exon in order to assess statistical significance. Box widths are proportional to the number of probes in each class and position tested. Signals in exon/intron sets (marked with lines and symbols) are significantly different (*P < 2.2 × 10^-16^, ^§^*P *< 7.2 × 10^-7^, ^†^*P *< 5.6 × 10^-13^, ^‡^*P *< 0.0007, ^+^*P *< 6.7 × 10^-09^; Welch two sample *t*-test).

To our surprise, intronic regions showed distinct patterns with respect to the chromatin-related features. The overall H3 occupancy was lower in introns than in surrounding exons, and it dropped even lower in exon-intron junctions at both 5' and 3' ends of introns (Figure 2b). A pattern of decreasing nucleosome occupancy at exon-intron boundaries has also been described in other organisms [29,30]. Accordingly, the FAIRE signals were substantially higher in introns than in exons, similar to the promoter and terminator regions (Figures 2b and 5a). This effect of increased FAIRE signals was not dependent on intron position within genes (Figure 6b). The differential patterns were not caused by sequence bias between introns and exons because the hybridization signals were normalized using genomic DNA signals (or input signals for ChIP-chip experiments) to correct for hybridization differences due to GC content [7,31]. Moreover, within average introns, we observed higher Pol II and FAIRE signals towards the 3' ends of introns (Figure 2b). This effect did not reflect any sequence disparity: a GC content comparison of 25-bp sequences (the length of one microarray probe) at either end of introns revealed no significant differences (*p *= 0.48).

Finally, we also detected significantly lower densities of the H3K36me3 modification (normalized for histone H3 density) within introns compared to surrounding exons (Figures 2b and 5c). Other papers have also reported such differential marking of introns and exons for the H3K36me3 modification in worms and humans [12,29,30]. This modification is enriched within the ORFs of transcribed genes and is catalyzed by the histone methyltransferase Set2 [32,33], which is conserved in fission yeast [14]. The H3K36me3 modification depends on the interaction of Set2 and the terminus of Pol II [34]; it is possible that the altered transcription kinetics that we detect in intronic regions interferes with H3K36me3 marking. It has been reported that Pol II in human cells is more enriched in exons than in introns, the reverse from our data [29]. A possible explanation for this discrepancy is that, in the previous study, any signals that fall within a 400-bp window (centered on the exon) are associated with that exon. Given the small average size of human exons (approximately 200 bp), extended intronic sequences on either side of exons would have been included with the exons for the analysis. If Pol II pauses at the 3' end of introns, as indicated by the Pol II and FAIRE enrichments in the much smaller fission yeast introns (typically < 100 bp), this may not have been detected in human [29]. Of course, it is also possible that Pol II progression across exonic and intronic regions differs between fission yeast and human genes.

### Intronic transcription and chromatin are affected by gene expression levels

**Figure 7:**
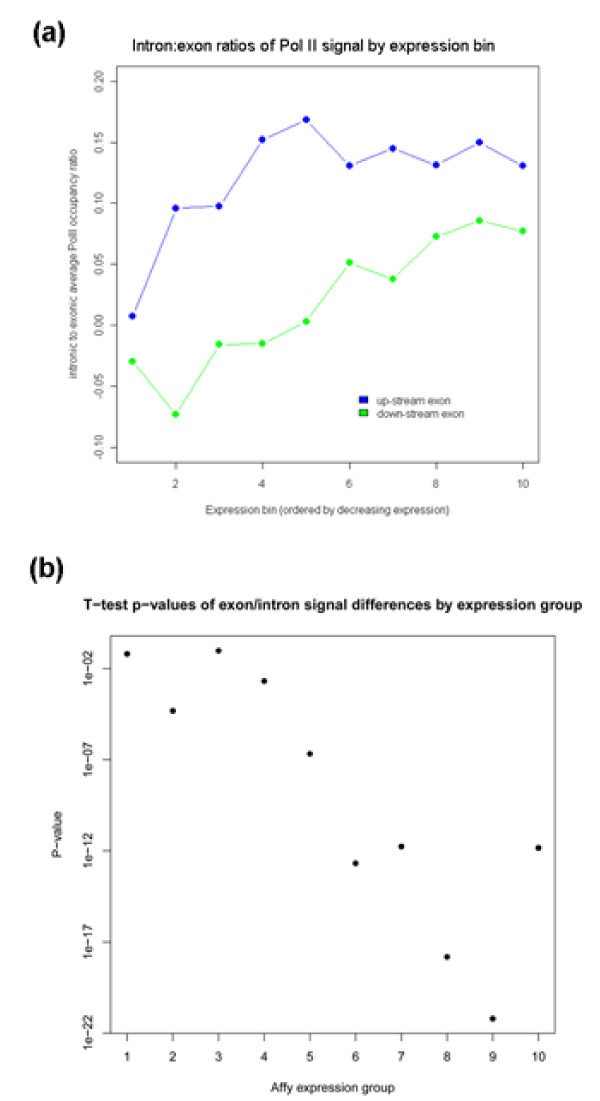
**Inverse relationship between gene expression and Pol II accumulation in introns**. **(a) **The ratios between average Pol II occupancy in upstream (blue) and downstream (green) exons relative to average Pol II occupancy in introns (in log_2 _values) are plotted as a function of expression bins. **(b) **Pol II occupancy signals from intron and exon probes for each expression group were used for a Welch two sample *t*-test, and the resulting *P*-value is plotted against the expression bins. The increasing significance of the *P*-values is inversely correlated with the gene expression level.

As described above for other gene regions, we also assessed the effects of transcript levels on the observed Pol II- and chromatin-related patterns across intronic regions (Figures 3 and 4). The introns of lowly expressed genes showed more pronounced drops in H3K36me3 modification signals relative to neighboring exons (Figure 3c). Strikingly, the relative difference in Pol II enrichments in introns compared to exons was directly related to the expression level of genes: the ratios of intronic to exonic Pol II occupancy levels increased with decreasing gene expression (Figure 7a). The same effect was evident when plotting the *p*-values of *t*-tests of the intron and exon signals for each expression bin against expression bin numbers (Figure 7b). These data, which cannot be explained by biased intron size as a function of gene expression (Additional file 2), demonstrate that with decreasing gene expression, there is decreasing H3K36me3 modification and increasing Pol II accumulation within intronic regions relative to exonic regions.

### Pol II enrichment in intronic regions

In accordance with the FAIRE signals, Pol II occupancy was also significantly higher on average in intronic regions than in exonic regions (Figures 2b and 5b). Consistent results were obtained from Pol II occupancy and quantitative real time PCR data of single genes (Additional files 1 and 3). This increased Pol II signal was not dependent on intron position within genes (Figure 6b). The average intron of spliced genes thus showed a pattern of Pol II enrichment similar to the promoter and terminator regions, raising the possibility that Pol II also accumulates in intronic regions. Notably, the Pol II and FAIRE signals increased throughout intronic regions and peaked towards the 3' ends of introns (Figure 3a, d). We therefore propose that Pol II actually accumulates at the 3' end of introns before resuming transcription. Given that Pol II accumulation was most pronounced in the most lowly expressed genes (Figures 3a and 7), any pausing seems to mostly affect genes that are poorly transcribed. Anti-sense transcription is unlikely to cause Pol II accumulation in introns as only 11 of 372 anti-sense transcripts actually overlap with introns and none reside entirely within introns [7].

Analyzing the processivity of Pol II, it has been noted that transcription does not continuously progress at the highest possible speed [35]. Pol II enrichment in introns could be related to observations that transcriptional speed can play a role in influencing alternative splicing of transcripts [36]. We envisage two, not mutually exclusive, possibilities why Pol II is enriched on introns. First, certain chromatin remodeling factors required to displace nucleosomes could be limiting. Recent studies have noted that exons contain well positioned nucleosomes relative to introns [29,30,37]. A sudden 'road block' of nucleosomes at the end of introns might cause Pol II to slow down or pause. Alternatively, or in addition, Pol II enrichment in introns could be directly linked to co-transcriptional splicing and could reflect the time required for splicing to finish before transcription can resume. Although we have not investigated the dynamics of the Pol II enrichment, evidence exists for a kinetic link between transcription and splicing [38], where cellular treatment to pause elongating Pol II results in increased co-transcriptional splicing. Some RNA processing or export factors are known to be associated with intronic regions [39]. Moreover, we have previously observed a global coordination between transcriptional and splicing efficiencies, with increased transcription leading to increased splicing in two genes tested [7].

## Conclusions

We conclude that intronic regions in fission yeast show patterns distinct from exonic regions with respect to several transcription- and chromatin-related features analyzed here, and that these patterns are related in large part to the transcriptional activities of genes. Furthermore, our data suggest that Pol II accumulates at the 3' end of introns, most notably in lowly expressed genes.

Intriguing studies in budding yeast have recently reported splicing-related pausing of Pol II during transcription [40-42]. Carillo Oesterreich *et al*. [42] found that Pol II pauses after the last intron to allow sufficient time for splicing before transcriptional termination. Alexander and co-workers [41] demonstrate that Pol II accumulates transiently at the 3' ends of introns on two reporter genes, which coincides with splicing factor recruitment and the detection of spliced mRNA. This pausing is tied to productive splicing and is accompanied by phosphorylation of the paused Pol II. The authors propose that transcriptional pausing is enforced by a checkpoint that is linked to co-transcriptional splicing [41].

Our data confirm and extend these findings in several respects. First, we provide evidence for intronic Pol II enrichment in fission yeast, which is only distantly related to budding yeast and contains many more introns (approximately 5,000 versus approximately 300 introns), suggesting that this phenomenon is conserved throughout eukaryotes. Second, we provide global data for all genes and introns, indicating that Pol II enrichment in introns is a general phenomenon. Third, we show that Pol II enrichment is linked to gene expression levels: the relative difference in Pol II enrichment in introns compared to exons is most pronounced in the lowly transcribed genes and becomes weaker in more highly transcribed genes. Moreover, the lowly transcribed genes also show the largest drop in H3K36me3 modification within intronic regions. These findings are consistent with differential H3K36me3 marking of intronic regions reflecting disrupted local chromatin structure caused by Pol II accumulation and splicing, which could interfere with H3K36me3 marking by Set2. On the other hand, it is possible that the differential H3K36me3 marking provides a favorable chromatin context for splicing to occur.

The global coordination between transcriptional and splicing efficiencies [7] and the inverse relationship between Pol II pausing and gene expression levels have important implications for current models of transcription and splicing. We propose that highly expressed genes out-compete lowly expressed genes for limiting splicing factors, leading to increased Pol II accumulation in the introns of lowly expressed genes. Transcription has been shown to take place in 'transcription factories' [43-46], and we speculate that only the highly transcribed genes are embedded in the processive environments of such factories, where abundant processing and splicing factors promote effective intron splicing and thus transcriptional elongation. Recent findings in fission yeast reveal that highly expressed genes associate with each other in the nucleus [47]. So if actively expressed genes either create, or are recruited to, highly processive transcription factories, all the steps required to generate mature mRNAs could be completed more efficiently and in a coordinated manner. Further investigations will define the precise mechanisms of the striking coordination between transcription, chromatin and splicing, and the functional importance of Poll II pausing within introns.

## Materials and methods

### Yeast strains and experimental conditions

Wild-type fission yeast cells (972 *h^-^*) were grown in rich yeast extract media at 32°C before being harvested for all experiments at exponential phase (approximately 5 × 10^6 ^cells/ml).

### ChIP-chip methods

Chromatin immunoprecipitions were performed, in biological duplicate, as described [7] using an antibody specific for the Pol II carboxy-terminal domain (CTD) (4H8, Abcam Cambridge, UK), histone H3 (ab1791, Abcam) or H3K36me3 (ab9050, Abcam). The two Pol II ChIP-chip experiments analyzed here were those reported by [7]. The whole cell extract was prepared using a Fastprep machine with glass beads to break cells after fixation, and the resulting lysate was sonicated to an average size of approximately 150 bp using the bioruptor (3 × 5', 30 s on, 30 s off). The immunoprecipitated material and input control were amplified in two steps as described [48]. During the second step, dUTPs were added to the PCR mix for subsequent fragmentation of the products. Fragmentation and labelling of the amplified products were performed using the GeneChip^® ^WT Double-Stranded DNA terminal labelling kit (Affymetrix Santa Clara, CA, USA). The duplicated immunoprecipitated samples and corresponding input material were hybridized on four separate Affymetrix GeneChip^® ^*S. pombe *Tiling 1.0FR arrays. The log_2 _signals of the probes on the input arrays were subtracted from the log_2 _signals of the Pol II arrays and the biological replicates were averaged. The H3K36me3 signals were normalized for the histone H3 signals.

### FAIRE methods

Biological triplicates of FAIRE were performed essentially as described [15]. Briefly, yeast cells were fixed with formaldehyde in medium at a final concentration of 1%. Cells were left to incubate for 10 minutes at room temperature before being spun down, washed once with water, and resuspended in the same lysis buffer as for ChIP with protease inhibitors (mini-complete EDTA free tablets, Roche Applied Science, Welwyn, UK). Cells were broken using glass beads and a Fastprep machine (20 seconds at 6.0 m/s) and then sonicated using a Bioruptor (Diagenode, Liège, Belgium) with 6 minutes total time (15 s on, 30 s off). DNA was phenol/chloroform extracted twice, and the resulting material was RNAse treated for 20 minutes before re-precipitating. The resulting DNA was then labeled according to standard Affymetrix protocols. The log_2 _FAIRE signals were normalized by subtracting the average signal of three genomic DNA hybridizations to correct for GC bias.

### Probe mapping for bulk signal differences

For analyzing differences in Pol II, histone H3 and H3K36me3 ChIP or FAIRE signals in introns and exons, 25-bp Affymetrix probes were mapped back to the *S. pombe *genome (GeneDB). Probes where the entire 25 bp length fell within an intron or exon were classed as 'intron probes' or 'exon probes', respectively. All probes that fell entirely within the ORF of unspliced genes were used for calculating the signal of unspliced genes.

### Average gene calculations

Every annotated *S. pombe *gene (downloaded from GeneDB) was divided into three parts, the promoter, coding, and terminator regions; in the case of spliced genes, the ORF was further divided into exons and introns. For both unspliced and spliced genes, 400-bp regions upstream of the start of the ORF and downstream of the end of the ORF were taken as the promoter and terminator, respectively. These 400-bp windows were divided into ten bins of 40 bp each, and Affymetrix probes were assigned to bins depending on where their midpoint fell (13th base pair). For the ORFs of unspliced genes, the lengths of ORFs were divided into 20 bins of equal size, with Affymetrix probes being assigned to bins based on their midpoint position. For spliced genes, each intron and exon was first divided into 10 or 20 bins of equal size, respectively, with probes assigned to bins based on their midpoint. In order to calculate an average of every exon-intron-exon junction without counting probes multiple times, the last ten bins of every upstream exon, the ten bins of every intron, and the first ten bins of every downstream exon were used to average probe signals from the various experiments. Probes falling in the first ten bins of every first exon and the last ten bins of every last exon were averaged to create the first and last ten bins for upstream and downstream exons, respectively.

### Average gene calculations by expression group

Replicate gene expression data collected previously [7] from Affymetrix Yeast 2.0 Genechip^® ^arrays were first filtered for undetectable signal (< 1; 480 of 5,296 genes excluded) and then sorted into spliced and unspliced genes (2,218 and 2,598 genes, respectively). Lists of spliced/unspliced genes were then ranked in descending order and split into 10 equal groups (approximately 220 and 260 genes per group for spliced and unspliced genes, respectively). Average gene profile calculations were then performed as described above for genes within each expression bin.

### Accession numbers

All microarray data used have been submitted to ArrayExpress under the accession number E-TABM-946.

## Abbreviations

bp: base pair; ChIP-chip: chromatin immunoprecipitation on microarray; FAIRE: formaldehyde-assisted isolation of regulatory elements; H3K36Me3: lysine 36 trimethylation of histone H3; ORF: open reading frame; PCR: polymerase chain reaction; Pol II: RNA polymerase II.

## Authors' contributions

BTW, SM and JB designed and supervised the research and discussed the results; BTW, SM, and SC all performed experiments with help from SW and SA. BTW analyzed the data with the help of JB and SM and drafted the manuscript with revisions by SM and JB. All authors have read and approved the final manuscript.

## Supplementary Material

Additional file 1 **Single-gene examples of Pol II occupancy**. **(a-f) **Affymetrix tiling array data for RNA Pol II is shown for three genes (SPAC13G7.11 (a), SPBC1773.01 (b), SPCC126.05c (c)) with low expression (ranked 2,447,1,666, and 2,310 out of 4,816, respectively, according to Affymetrix expression data) and three genes (SPBC4F6.18c (d), SPAC17G6.06 (e), SPCC24B10.09 (f)) with high expression (ranked 216, 90, and 56 out of 4,816, respectively, from data as above). Additional annotated features are shown (expression rankings are SPAC13G7.12c (3,307), SPBC1773.02c (2,764), SPCC126.04c (2,123), SPCC126.06 (2,591), SPBC4F6.17c (1,944), SPAC17G6.05c (4,227), SPAC17G6.07c (811), SPCC24B10.08c (3,300), SPCC24B10.10c (3,376)) and the range of absolute values of RNA Pol II signals (as previously calculated [7]) are shown on the left side of each panel. Introns within genes shown are indicated by red lines. Click here for file

Additional file 2 **Expression level of spliced genes is not biased by intron size**. A scatterplot showing the size of each intron in the annotated *S. pombe *genome and the corresponding gene expression level (according to previously published Affymetrix microarray data [7]). Click here for file

Additional file 3 **Validation of Pol II occupancy in single genes by quantitative PCR**. Click here for file
