# Supplementary material for: Differential patterns of intronic and exonic DNA regions with respect to RNA polymerase II occupancy, nucleosome density and H3K36me3 marking in fission yeast
Source: Genome Biol. 2011 Aug 22;12(8):R82. doi: 10.1186/gb-2011-12-8-r82 (PMC3245620; doi:10.1186/gb-2011-12-8-r82)
Supplement: Additional file 3 — Validation of Pol II occupancy in single genes by quantitative PCR. [file gb-2011-12-8-r82-S3.PDF]

<u>Additional file 3</u>

Single gene validation of Pol II enrichment by qPCR

qPCR was performed on both Pol II IP and input material that was prepared as described in the main methods section. qPCR was carried out using the SYBR green as detailed herafter. SYBR green qPCR reactions were performed using 5ng of DNA, 5 µl of the Fast SYBR Green Master Mix (Applied Biosystems) and 2 µM of each primer in a total volume of 10 µl. Melting curves were performed using dissociation curve software (SDS 2.2.2) to ensure only a single product was amplified. The ABI PRISM® 7900HT (Applied Biosystems) was used to detect the amplification level and was programmed with an initial step of 3 minutes at 95˚C, followed by 40 cycles of: 5 sec at 95˚C and 30 sec at 60˚C. All reactions were run in triplicate and the average values of Cts were used for quantification. qPCR reactions were performed using the primers listed in Supplemental table 1 and the results are summarized in Supplemental table 2.

Analysis of Pol II enrichment levels was calculated as the relative difference in threshold cycle (Ct) between input and ChIP material for various regions within each gene for each primer pair. The difference in Ct for ChIP and input was smallest (e.g. greatest relative enrichment) for intron regions in 5 of 8 cases, and slightly higher than the promoter region for 2 of the lowly expressed genes.

<u>**Additional table 1 - Primers used for qPCR**</u>

>SPBC1709.12-intron5'
ttgtgcagctacgtgaatcaaagtgg
>SPBC1709.12-intron3'
ctacccatctgtattgcagttagtatg
>SPBC1709.12-prom5'
caaccgttccgcgtattagcctaa
>SPBC1709.12-prom3'
gggagggctggtatatattaaaatgg
>SPBC1709.12-exon3'
ggttgctcgtaacatggtttttttacgca
>SPBC1709.12-exon5'
cttctgaagatgaaatgccacaatg
>SPBC1709.12-term5'
taagcaggcacttacaaatctagtca
>SPBC1709.12-term3'
tccagtttgcacaagtttgtatggcc

>SPAC1805.11c-term5'
gcagccaccatccagcttcatcc
>SPAC1805.11c-term3'
aggttaaccctactgccgttgcca
>SPAC1805.11c-exon5'
tggcaacggcagtagggttaacct
>SPAC1805.11c-exon3'
catggttgaaactgccgccattcg
>SPAC1805.11c-intron5'
tacctaaatatgtgtcagttcattgt
>SPAC1805.11c-intron3'
atccgtaagttcaacttaaatgattc
>SPAC1805.11c-prom5'
ggtggtgcagaggaaaagtttcacc
>SPAC1805.11c-prom3'
tcccaacaccgcacagtatttacc

>SPBC409.12c-term5'
cccatgtgtacttattgatctgtttcc
>SPBC409.12c-term3'
cacattgacgcggagatttaattgcc
>SPBC409.12c-exon5'
ccgcccatgttgtagtaatgtttg
>SPBC409.12c-exon3'
gcaaaagagttgcacctcccacttg

>SPAC15e1.03-term5'
cggtgatcctaaaagcaattcaaagt
>SPAC15e1.03-term3'
gagaacagaccttagcgttagcg
>SPAC15e1.03-exon5'
caagaagggacctgattccaagct
>SPAC15e1.03-exon3'
cgaaggacaaccttcttggtaacc

>SPBC409.12c-intron5'
gggagctatagaggcagatgcttctc
>SPBC409.12c-intron3'
ggtaacgaatatcagcattgaggca
>SPBC409.12c-prom5'
ggtcattcccttaaagactttcaacc
>SPBC409.12c-prom3'
cattgcttaattttcgaaaacgctgcg

>SPCC61.01c-intron5'
gagactaaaagagaagacattgtgttag
>SPCC61.01c-intron3'
tgggtatgttagtattgtagtcatgat
>SPCC61.01c-prom5'
catccagagtgtatggtaaatggcag
>SPCC61.01c-prom3'
gtttaccaacttcattaaacatctcgg
>SPCC61.01c-exon3'
cgagtcaggtagtttcaccagactc
>SPCC61.01c-exon5'
gcgaaaacgggatagcgtgcatatt
>SPCC61.01c-term5'
gtcgtaatcgtactccaatgaccca
>SPCC61.01c-term3'
agaatagtgaacctaagtatgctatcc

>SPAC20G8.05c-intron5'
cataccgaacccttcggttcattac
>SPAC20G8.05c-intron3'
ctaccttaagcgctacgatgattcaag
>SPAC20G8.05c-prom5'
gtgatttgttgttggtaaattcctggt
>SPAC20G8.05c-prom3'
gcactcagataggcaacggttgct
>SPAC20G8.05c-exon3'
gttcgccaacacgtagcgcttttc
>SPAC20G8.05c-exon5'
agaaggaacgggaatagaaagttca
>SPAC20G8.05c-term5'
ttctggtaattatcggtcggcttc
>SPAC20G8.05c-term3'
acacgatgatgaatttgcctgctc

>SPAC15e1.03-intron5'
ggtatgtcgaagaatgctgataacga
>SPAC15e1.03-intron3'
ggatttgagaggtattcaaccacggc
>SPAC15e1.03-prom5'
gcgttgaacgacattacagcagca
>SPAC15e1.03-prom3'
gtgatggttagatggtggtgattagg

>SPBC1685.10-term5'
gtcttgttcatatcttgcatgtcttg
>SPBC1685.10-term3'
ggtcaaacaataccaacaacgtgcaa
>SPBC1685.10-exon5'
gatgtcaagtgccctggatgcttc
>SPBC1685.10-exon3'
gaacaagaccttttagttctttcttctg
>SPBC1685.10-intron5'
aattctgtactttcgagaataaactgtt
>SPBC1685.10-intron3'
gcaatgagtctttgagttcaaacca
>SPBC1685.10-prom5'
gtgctgcactatttacgagtaacaa
>SPBC1685.10-prom3'
cttgttagtgaattgctcggggta

>SPBC119.02-term5'
cgcaatctagagtttgtttctgtgttg
>SPBC119.02-term3'
ctttactagctacattatcgccatccc
>SPBC119.02-exon5'
catcccaacatcaattcaaacggtagc
>SPBC119.02-exon3'
gtagacgtgcgcaatttcaggcaca
>SPBC119.02-intron5'
aggtatgtaactatttagtcttgtgtag
>SPBC119.02-intron3'
gcatcactgtcaaccataccgatt
>SPBC119.02-prom5'
gcgccatcctgaaaaattgcttactc
>SPBC119.02-prom3'
gccatcgttactggtttgcttttgcg

Additional table 2 – qPCR results for 4 regions within highly or lowly expressed genes

Lowly expresed genes

Region	Region	Sample	Avg Ct	Stdev	
promoter	SPBC1709.12	CHIP	27.90	0.30	3.45
		INPUT	24.45	0.05	
exon		CHIP	28.43	0.19	3.44
		INPUT	24.99	0.03	
intron		CHIP	27.64	0.26	3.10
		INPUT	24.53	0.21	
terminator		CHIP	28.67	0.06	3.41
		INPUT	25.26	0.24	
promoter	SPBC409.12c	CHIP	28.69	0.02	3.83
		INPUT	24.86	0.10	
exon		CHIP	28.28	0.44	4.16
		INPUT	24.12	0.15	
intron		CHIP	29.79	0.28	4.05
		INPUT	25.74	0.17	
terminator		CHIP	28.70	0.18	4.17
		INPUT	24.53	0.09	
promoter	SPCC61.01c	CHIP	28.69	0.18	3.99
		INPUT	24.71	0.11	
exon		CHIP	36.50	0.16	4.39
		INPUT	32.11	0.28	
intron		CHIP	27.76	0.17	3.34
		INPUT	24.42	0.09	
terminator		CHIP	29.03	0.25	3.80
		INPUT	25.23	0.16	
promoter	SPAC20G8.05c	CHIP	28.12	0.24	3.22
		INPUT	24.91	0.11	
exon		CHIP	28.32	0.27	3.34
		INPUT	24.97	0.06	
intron		CHIP	28.96	0.15	3.33
		INPUT	25.64	0.08	
terminator		CHIP	28.40	0.16	3.50
		INPUT	24.90	0.08	

Ct difference relative to intron					Average difference
promoter difference	0.34	-0.22	0.65	-0.11	0.26
exon difference	0.34	0.11	1.05	0.02	0.50
terminator difference	0.30	0.12	0.46	0.18	0.30

Highly expressed genes

Region	Region	Sample	Avg Ct	Stdev	
promoter	SPAC1805.11c	CHIP	28.31	0.08	2.36
		INPUT	25.95	0.09	
exon		CHIP	28.76	0.10	2.32
		INPUT	26.44	0.09	
intron		CHIP	29.50	0.24	2.07
		INPUT	27.44	0.52	
terminator		CHIP	28.59	0.31	2.20
		INPUT	26.39	0.13	
promoter	SPAC15E1.03	CHIP	27.75	0.09	2.54
		INPUT	25.21	0.24	
exon		CHIP	27.13	0.20	1.96
		INPUT	25.17	0.18	
intron		CHIP	26.42	0.14	1.53
		INPUT	24.89	0.08	
terminator		CHIP	26.51	0.13	1.87
		INPUT	24.63	0.25	
promoter	SPBC119.02	CHIP	28.68	0.30	2.89
		INPUT	25.79	0.20	
exon		CHIP	28.35	0.21	2.63
		INPUT	25.72	0.08	
intron		CHIP	27.13	0.20	2.21
		INPUT	24.93	0.17	
terminator		CHIP	27.54	0.22	2.85
		INPUT	24.69	0.07	
promoter	SPBC1685.10	CHIP	26.55	0.17	1.97
		INPUT	24.58	0.18	
exon		CHIP	27.96	0.11	1.96
		INPUT	26.00	0.33	
intron		CHIP	26.97	0.23	2.12
		INPUT	24.85	0.23	
terminator		CHIP	28.08	0.19	1.95
		INPUT	26.13	0.14	

Ct difference relative to intron					Average difference
promoter difference	0.30	1.01	0.68	-0.15	0.66
exon difference	0.25	0.43	0.42	-0.16	0.37
terminator difference	0.13	0.34	0.64	-0.17	0.37
